# A teenage girl with an untreatable nephrotic syndrome: Answers

**DOI:** 10.1007/s00467-020-04708-y

**Published:** 2020-07-27

**Authors:** Marta Cognigni, Marco Pennesi, Giulia Pennesi, Egidio Barbi

**Affiliations:** 1 University of Trieste, Trieste, Italy; 2 Institute for Maternal and Child Health—IRCCS Burlo Garofolo, Trieste, Italy; 3 Edinburgh Napier University, Edinburgh, UK

1. The MRI showed the presence of a mediastinal mass suggestive of a lymphoma, which was diagnosed as Hodgkin’s lymphoma following a lymph node biopsy. This finding confirmed the suspicion of a paraneoplastic glomerulonephritis (PG).

Paraneoplastic glomerulonephritis is an uncommon complication of both solid and haematological malignancies, with glomerular lesions induced by products from tumour cells rather than being directly related to tumour invasion or metastasis [1]. Though PG is quite rare, particularly in childhood and adolescence, it still may be quite easily mistaken for idiopathic glomerulonephritis due to the delayed diagnosis of malignancy. About 11% of adult patients with glomerulopathies, generally presenting as nephrotic syndrome (NS), have cancer and in two-thirds of the cases, NS precedes the diagnosis of malignancy [2]. This case exemplifies one of the various glomerular lesions that can occur in different forms of PG. The link between haematological malignancies and NS is mostly observed in Hodgkin’s lymphoma. The incidence of this association is 0.4% in the adult population [3], while it is not well known in childhood: two small series described an incidence rate between 0.6 and 1% [4, 5]. The most frequent observed renal lesion associated with Hodgkin’s lymphoma is minimal change disease (1%), while the occurrence of FSGS is much rarer [6]. According to this study [7], PG usually does not run parallel to the course of the malignancy as it usually appears before the cancer is diagnosed. This clinical observation has also been confirmed in childhood [4]. The majority of patients with PG secondary to lymphoid malignancies showed systemic symptoms (fever, weight loss, arthralgia) and high inflammatory laboratory parameters (CRP, ESR, fibrinogen), indicating that these findings should be used as red flags to promptly recognise an atypical NS. PG can also occur in solid tumours, although its incidence and prevalence in association with solid malignancies remain unclear and the causal link is still unknown. The most common solid tumour–associated glomerulopathies are carcinoma of the lung and of the gastrointestinal tract, usually associated with membranous nephropathy [8].

2. Paraneoplastic glomerulonephritis needs to be distinguished from idiopathic kidney disease. The recognition of PG before the detection of malignancy requires a high index of suspicion and needs to be included in the differential diagnosis of secondary NS, even in children and adolescents. A work-up for malignancy should be considered in all patients presenting an apparently idiopathic NS showing a poor response to steroid or immunosuppressive therapy. An atypical age of onset, the presence of systemic symptoms (persistent fever, arthralgia), persistent positive acute reactant phase laboratory tests and persistent proteinuria without other features of NS should further strengthen the suspicion of an occult lymphoma [6, 7].
3. The effective treatment of the tumour is reported to result in the remission of the NS without the need for a specific therapy directed at the kidney lesion [4]. Hence, the prognosis of these patients is good, with NS disappearing after cancer treatment. Remarkably proteinuria often reappears in association with eventual relapses of malignancy [9] and could therefore be the first sign of the tumour’s recurrence. Cancers associated with NS are reported to have a worse prognosis in the adult population. The 1-year mortality rate in cancer patients with preexisting NS is higher than in cancer patients without prior NS, particularly, in those with haematological malignancies [10].

## Patient outcome

The remission of NS was attained within 3 months after the initiation of chemotherapy. During the last 4 years of follow-up, proteinuria did not relapse and neither did the lymphoma.

## Discussion

Paraneoplastic glomerulonephritis is a rare occurrence in children with solid and haematological tumours. Prolonged proteinuria, an atypical age-onset for minimal change disease, ineffective steroid, and/or immunosuppressive therapy, presence of systemic symptoms, and persistence of laboratory alterations should all suggest a paraneoplastic syndrome. Adequate and effective treatment of the tumour will result in remission of the NS. Even if the diagnosis of PG does not run parallel to the tumour, the reappearance of NS correlates directly with the tumour’s relapse. For this reason, the monitoring of proteinuria becomes relevant to ensure early detection of malignancy recurrence.
